# A Comparison of Deformed Wing Virus in Deformed and Asymptomatic Honey Bees

**DOI:** 10.3390/insects8010028

**Published:** 2017-03-07

**Authors:** Laura E. Brettell, Gideon J. Mordecai, Declan C. Schroeder, Ian M. Jones, Jessica R. da Silva, Marina Vicente-Rubiano, Stephen J. Martin

**Affiliations:** 1 School of Environment and Life Sciences, The University of Salford, Manchester M5 4WT, UK; s.j.martin@salford.ac.uk; 2 Viral Ecology, Marine Biological Association, Plymouth PL7 5BU, UK; gidmor@MBA.ac.uk (G.J.M.); dsch@MBA.ac.uk (D.C.S.); 3 School of Biological Sciences, University of Reading, Reading RG6 6AJ, UK; i.m.jones@reading.ac.uk; 4 Department of Earth, Ocean & Atmospheric Sciences, The University of British Columbia, Vancouver, BC V6T 1Z4, Canada; 5 Centro de Ciências Agrárias, Ambientais e Biológicas, Universidade Federal do Recôncavo da Bahia, Rua Rui Barbosa, 710 Centro, Cruz Das AlmasBahia State 44380-000, Brazil; r.jessicarosa@gmail.com; 6 VISAVET, Faculty of Veterinary Science, Complutense University de Madrid, 28040 Madrid, Spain; mvrubiano@vet.ucm.es; 7 Animal Health Department, Faculty of Veterinary Science, Universidad Compultense de Madrid, 28040 Madrid, Spain

**Keywords:** deformed wing virus, honeybee, Varroa, next generation sequencing, RTPCR

## Abstract

Deformed wing virus (DWV) in association with *Varroa destructor* is currently attributed to being responsible for colony collapse in the western honey bee (*Apis mellifera*). The appearance of deformed individuals within an infested colony has long been associated with colony losses. However, it is unknown why only a fraction of DWV positive bees develop deformed wings. This study concerns two small studies comparing deformed and non-deformed bees. In Brazil, asymptomatic bees (no wing deformity) that had been parasitised by Varroa as pupae had higher DWV loads than non-parasitised bees. However, we found no greater bilateral asymmetry in wing morphology due to DWV titres or parasitisation. As expected, using RT-qPCR, deformed bees were found to contain the highest viral loads. In a separate study, next generation sequencing (NGS) was applied to compare the entire DWV genomes from paired symptomatic and asymptomatic bees from three colonies on two different Hawaiian islands. This revealed no consistent differences between DWV genomes from deformed or asymptomatic bees, with the greatest variation seen between locations, not phenotypes. All samples, except one, were dominated by DWV type A. This small-scale study suggests that there is no unique genetic variant associated with wing deformity; but that many DWV variants have the potential to cause deformity.

## 1. Introduction

Honey bees with deformed wings have become a universal sign for the presence of deformed wing virus (DWV) in colonies infested by *Varroa destructor* across the world. DWV is reported as the most important honey bee viral pathogen causing the death of millions of colonies across the northern hemisphere [1,2]. However, the proportion of honey bees with deformed wings (i.e., symptomatic bees) in a colony is normally low (<1%) despite a high proportion of asymptomatic honey bees being infected with high viral titres of DWV [3]. This is, in part, due to symptomatic bees dying as pupa or within 48 h of emerging from their brood cell [4]. Although normally low, up to 66% of individuals can have wing deformity in a severely infected colony [5], but these levels are rarely seen.

Wing deformity was originally believed to be caused by the removal of the developing bees’ haemolymph by the mites’ feeding activities [6]. Although deformed wings can occur due to insufficient nutrition or fluids [7], there was a noticeable increase in the number of deformed bees associated with Varroa infested colonies, which was later linked to the ability of Varroa to transmit DWV to developing honey bees [7]. On the isolated island of Fernando de Noronha Varroa, mites have been feeding on its honey bees for the past 32 years; unique to this population, DWV has remained a low level covert infection. No bees with deformed wings have ever been recorded on the island [8,9,10], indicating that the mites’ feeding activity does not directly cause wing deformity. However, although both deformed and asymptomatic bees can have very high viral titres (×10^9^) [11], deformed bees have consistently higher DWV titres than asymptomatic bees [12,13]. It is not unknown for deformed bees to contain lower than expected DWV titres (×10^4^–×10^7^) [14]; this is a very rare occurrence and could be a result of a different, external factor. It was suggested by Gisder et al. [15] that the development of deformed wings was due to viral replication within the mite leading to a higher delivery into the bee and that this may not occur in most mites. Furthermore, DWV appeared to be present in the heads of deformed bees but only present in the thorax and abdomen of asymptomatic bees [16]. However, subsequent studies have found DWV also in the heads of asymptomatic bees, helping explain changes in their behaviour [17] and effects on their learning [18]. However, there is no specific, proven etiology for the disease and the pathogenesis, and cytopathology of DWV has yet to be directly studied. Alternate causes have been suggested: that deformity may arise as a consequence of the bees’ immune response to mite feeding [19], or that microbial septicaemia occurs as a result of microorganisms transmitted by Varroa [20], but the weight of evidence especially from the Fernando de Noronha study [10], currently does not support these mechanisms [15,16,21,22].

DWV is a quickly evolving group of closely related viruses [23], which is commonly referred to as a quasispecies [24]. This is made up of three master variants. Martin et al. [2] initially classified DWV as being composed of two master variants, type A which consists of DWV and Kakugo virus (KV) [3,17] and type B which refers to the Varroa destructor virus-1 (VDV-1) which was first isolated from Varroa [25], and was suggested to cause wing deformity [25,26]). Recently, type C, a third distinct variant, has been discovered in asymptomatic bees collected in Devon, UK [27]. Both types A and B are associated with disease symptoms [26,28], and are known to form recombinants [26] but the type A variant is more commonly associated with infestation by Varroa and subsequent colony collapse [2,29]. Conversely, the dominance of type B in a population has recently been shown to prevent the virulent type A becoming established and causing colony losses [30].

Recent work in colonies that have never been exposed to Varroa have shown that DWV consists of a wide diversity of variants, but that transmission by Varroa causes the amplification of dominant DWV variants and a major reduction in the subsequent virus diversity in the honeybee [2,31]. Further experimental manipulations have shown that this reduction in variant diversity occurs within the bee, not the mite [31]. These studies were conducted using asymptomatic bees. The quasispecies theory of viral evolution [32] may help to explain why only a small proportion of the bees become deformed, since a particular DWV variant that is able to reproduce rapidly in both mites and bee pupae may exist within the quasispecies infecting deformed bees, whereas a different variant could dominate in asymptomatic bees. Alternatively, the lack of a dominant variant but high viral diversity in asymptomatic bees could be hypothesised as the reason for the lack of development of the deformed wing phenotype, but given the current data this seems less likely.

The aim of this study was to use RT-qPCR, High resolution melt (HRM) and next generation sequencing (NGS) to determine if a particular variant was associated with wing deformity in honey bees parasitised by the Varroa mite.

## 2. Materials and Methods

### 2.1. Honeybee Samples

For the RT-qPCR viral quantification and wing deformity study, honey bees were collected from an apiary maintained by Universidade Federal do Recôncavo da Bahia (UFRB), Cruz das Almas (12.67° S, 39.1019° W), Bahia state, Brazil. We confirmed that the honey bees from this population were infected by the type A variant of DWV (Supplementary Figure S1) as were the bees from Hawaii [2]. For the NGS study, we used previously collected samples of honey bees from Hawaii (collected in 2012). Three pairs of samples were chosen, each consisting of a single deformed bee and a pool of 30 asymptomatic bees from the same colony. Each colony had been exposed to Varroa infestations for different lengths of time (Oahu = 5 years, Big Island East = 4 years, Big Island South = 3 years). The rarity of bees with deformed wings in both populations made it impossible to compare similar numbers of symptomatic and asymptomatic bees. The vast majority of adults with deformed wings contain high DWV loads ([13], this study), whereas DWV titres in individual asymptomatic bees are more variable [33], hence we used a pooled sample for the NGS study to ensure sufficient DWV genomes were present for sequencing.

### 2.2. Effect of Viral Load on Wing Deformity

**Figure 1:**
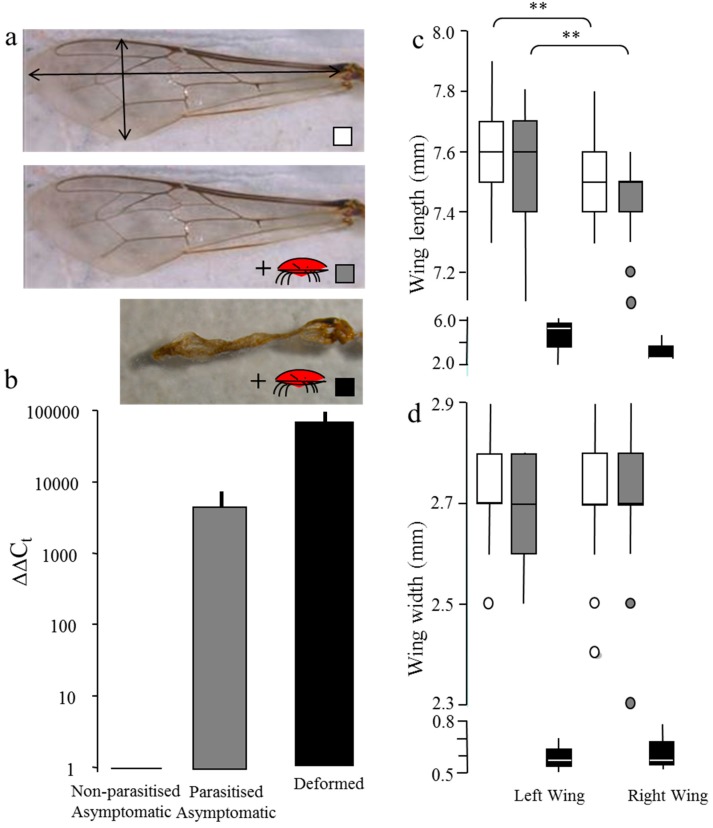
(**a**) Shows images of a normal forewing from a non-parasitised, parasitised asymptomatic, and a deformed honey bee; (**b**) Deformed wing virus (DWV) load of non-parasitised, parasitised asymptomatic, and deformed bees quantified by DWVQ RT-qPCR. Delta delta Cycle threshold (C_t_) values normalised against an Actin gene control and non-parasitised asymptomatic bees; [11] and relative to the non-parasitised asymptomatic bees, shown on a log scale (**c**) wing length and (**d**) width of 45 non-parasitised (clear box blots), 45 parasitised (grey box plots) and three bees with deformed wings (black box plots). Note the broken axis to deal with the large size differences between deformed and normal forewings. ** *p* < 0.001 between right and left wings.

In January 2015, in Brazil, a frame of an emerging brood was removed from three study colonies. Each emerging bee, along with the cell that it was emerging from, was checked for the presence or absence of Varroa mites. A total of 45 parasitised and 45 non-parasitised newly emerged worker bees were collected from the three frames. Only bees seen emerging from a cell were used. However, no emerging deformed bees were found despite 500 bees emerging from the frames. As such, a visual search of the three study colonies was conducted that resulted in just three deformed bees being located. As the vast majority of deformed bees develop from parasitised pupae [34], it is likely that these individuals emerged from infested cells, which is supported by the high DWV titres we detected (Figure 1). All bees were killed by freezing at −20 °C before their forewings were removed and mounted on a glass slide for morphometric analysis. Individual bees were then labelled with a unique label and shipped to the UK in a Dry Vapour Shipper at −186 °C for viral analysis. Each forewing (length and width) was measured using a Leica binocular microscope (×10) magnification fitted with a Leica camera. As these were newly emerged bees, no wing wear was present. As directional asymmetry in wing size in honey bees is well established [35,36], we measured both wings of the parasitised and non-parasitised groups and compared the results using a Mann–Whitney U test since not all wing measurement distributions were normally distributed.

For the RT-qPCR analysis, a random subset of ten parasitised and ten non-parasitised newly emerged asymptomatic bees were chosen along with the three deformed bees. Then each of the 23 individual bee samples was ground in liquid Nitrogen to a fine homogeneous powder and 30 mg material used for RNA extraction using the RNeasy mini kit (Qiagen, Venlo, The Netherlands), according to the manufacturer’s instructions. Total RNA samples were quantified using a Nanodrop 8000 (Thermo Scientific, Waltham, MA, USA). One microgram of isolated RNA was treated with DNase I (Promega, Madison, WI, USA), followed by Nanodrop quantification to standardise the amounts of total RNA to 25 ng/µL, before storage at −80 °C.

Total RNA was analysed in duplicate for each sample using the one-step SensiFAST SYBR No ROX One-step kit (Bioline, London, UK). RT-qPCR reactions contained 50 ng RNA, 1× SYBR one-step Sensimix, 2.5 mM MgCl_2_, 5 units of RNase inhibitor, and 7.5 pmol of each primer: DWVQ-F1 and R1 for DWV (primers bind within the RdRp gene) with Actin F1 and R1 as the reference gene [11] (Supplementary Table S1). Reactions were run on a Rotor-Gene Q Thermocycler (Qiagen) with an initial reverse transcription stage at 49 °C for 30 min and a denaturation step of 95 °C for 10 min, followed by 40 cycles of denaturation for 15 s at 95 °C, annealing for 30 s at 54 °C for DWV, and 58 °C for Actin, and extension for 20 s at 72 °C. The SYBR green signal was measured on the green channel after each extension step. A final dissociation melt curve was performed between 65 °C and 95 °C, at 0.5 °C increments, each with a 10 s hold and acquisition to the green channel. The melt curve was used to ensure that a single targeted product was amplified, and that no contamination was present in the reverse transcription negative controls or in the no-template controls. The threshold cycle (C_t_) value was determined for each sample using the QIAGEN Rotor—Gene Q Series Analysis software. All samples were run in duplicate and the average was taken. Those samples which had a standard deviation of ≥1 C_t_ were re-run to obtain duplicates with standard deviation <1 C_t_. Each sample was normalised against Actin, and then presented relative to the asymptomatic non-parasitised bees as ΔΔC_t_ values. Statistical differences were calculated using a pair wise Mann–Whitney U test when the data were not normally distributed.

### 2.3. Next Generation Sequencing, Assembly and Data Normalisation

RNA was again extracted from 30 mg of material from each of the six Hawaiian samples (three colonies that each contained a single deformed bee and 30 asymptomatic bees) using the RNeasy mini kit (see above). Total RNA was used for a cDNA amplification step using oligo dT priming followed by sequencing. Illumina sequencing (Hi-Seq 100 bp paired end reads) was carried out by The Earlham Institute, Norwich. A Bioinformatics pipeline designed to accommodate the large amount of variation found within DWV, first described in Mordecai et al. [30] was applied. This involved using reads which mapped to a custom BLAST database of DWV master variants type A (NC_004830.2 and Kakugo virus NC_005876.1), B (AY251269.2) and C (CEND01000001.1) database using an e value of 10e−05 to assemble DWV-like contigs using VICUNA which was specifically developed to deal with highly variable data. Read data were uploaded to the NCBI Sequence Read Archive under study number SRP095247.

**Figure 2:**
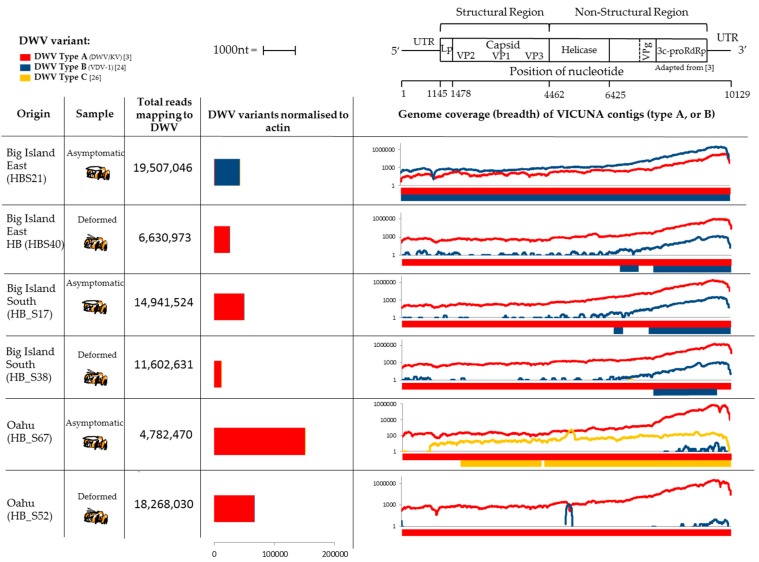
Genome coverage from the Illumina Hi-Seq data for the Hawaii colonies including a map of the DWV genome adapted from Lanzi et al. [3]. DWV type A, B and C genomes (in red, blue and yellow respectively) were assembled from the Illumina next generation sequencing (NGS) data from honeybees from Hawaii. DWV load was normalised to Actin. Breadth of genome coverage by Vicuna contigs is shown against the DWV genome for type A, B and C variants, as well as individual competitive alignment read depth coverage plots.

**Figure 3:**
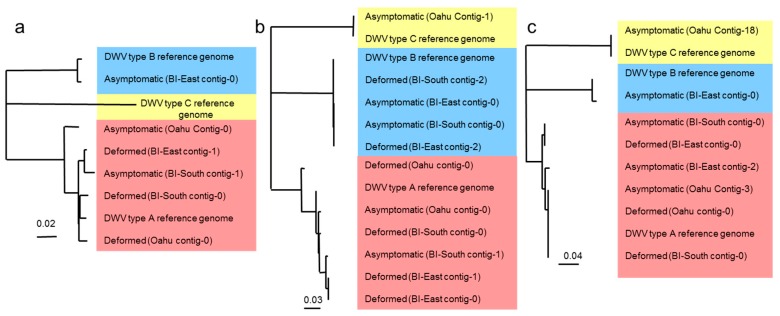
Phylogeny (neighbor joining) of type A, B and C Vicuna contigs covering the (**a**) 4360 bp region spanning the majority of the non-structural block; (**b**) RdRp region [11] and (**c**) Capsid region. DWV type A, B and C sequences are highlighted in red, blue and yellow respectively. The low diversity of type B sequences can be attributed to a low viral load. At no gene location do the deformed and asymptomatic form groups. BI = Big Island.

Viral contigs were imported into Geneious (Version 7.04, created by Biomatters, Aukland, New Zealand) and the “Map to Reference tool” was used to align the contigs against the DWV type A (NC_004830.2), B (AY251269.2) and C (ERS657949) reference genomes. These contigs were used to assess the breadth of genome coverage (Figure 2) as well as to analyse the dominant variants in each sample (Figure S3). The phylogenetic trees (Figure 3) were created within Geneious (Version 7.04, created by Biomatters) using a Tamura-Nei Genetic Distance model and a neighbor joining tree building method. In order to ensure that the contigs produced truly represented the viral populations, geneious competitive alignments were performed in which the raw sequencing read 1 files in fasta format were competitively aligned against DWV types A, B and C reference genomes (allowing for 5% mismatches and no gaps with reads with multiple best matches being discarded) to produce coverage graphs for reads corresponding to each type.

To quantify the number of DWV reads in each sample, the number of reads unambiguously mapping to each master variant were counted. These were then normalised against the number of reads that matched to part of the Actin gene used for RT-qPCR by Highfield et al. [11] using the geneious “map to reference” tool.

## 3. Results

### 3.1. Viral Quantification and Wing Deformity in Honey Bees

All 23 individual bees from Brazil tested positive for DWV using RT-qPCR. HRM analysis and Sanger sequencing indicated that all bees were dominated by the DWV type A (Supplementary Figures S1 and S2). The highest loads were consistently detected in the three bees with wing deformities (Figure 1b). This was followed by asymptomatic bees that had been parasitised by Varroa mites as pupae. The lowest DWV loads were detected in asymptomatic bees that had developed free from Varroa (non-parasitised bees). Due to the low viral load in the non-parasitised bees, primer dimer was also amplified along with the DWV RdRp diagnostic fragment and this would have led to an overestimate in viral load, so the actual amount may be lower than shown. Despite these significant differences in DWV load between the three groups, their wing morphology did not follow the same trend. The bees with deformed wings had the highest viral load, as expected. However, no significant differences in wing length (Left wing, U = 668, Z = 1.21, *p* = 0.22: Right wing, U = 709, Z = −1.53, *p* = 0.12) or wing width (Left wing, U = 694, Z = 0.91, *p* = 0.36: Right wing, U = 871, Z = 0.28, *p* = 0.78) were found (Figure 1c,d) between the asymptomatic, non-parasitised and parasitised groups of bees. In both groups, directional asymmetry was detected in wing length but not wing width. That is, the bees’ left wing was significantly longer than their right wing in both the non-parasitised (U = 493, Z = 3.71, *p* = 0.0002) and Varroa-parasitised (U = 402, Z = −3.52, *p* = 0.0004) groups (Figure 1c).

### 3.2. Next Generation Illumina Sequencing

Each of the six samples from Hawaii contained sufficient DWV reads to assemble into contigs that together covered the entire length of the genome (Figure 2). Five of the six samples were dominated by type A reads that yielded full genome coverage. However, a single asymptomatic sample from Big Island East was found to be dominated by type B reads that yielded full genome coverage of the type B genome (Figure 2). The breadth of type B coverage is less in the samples from Oahu where Varroa has been present for a long time where the type A variant dominates. The read depth coverage graphs for all samples showed a strong 3’ bias which can be attributed to the inherent 3’ bias of reverse transcription produced using oligo dT priming in the library preparation [37]. The plots confirmed that the asymptomatic sample from Big Island East was the only one that contained type B read coverage across the entire genome and that all others contained type B coverage at the 3’ end only (Figure 2). The samples from Oahu did contain low amounts of type B reads, however there was insufficient read depth for the assembler to produce contigs. The asymptomatic sample from Oahu contained a small number of DWV type C reads (*n* = 359). Although type C was not the dominant variant, coverage was sufficiently high to assemble contigs spanning the majority of the genome. Very low numbers of type C reads were found in other samples by counting reads unambiguously mapping to type C and were used when normalising DWV variants to actin (Figure 2), however the read depth was insufficient to generate type C contigs in any other sample. Although it is impossible to rule out that the DWV type C identified in this study is a result of contamination via barcode shifting originating from samples dominated by type C run on the same flow cell lane see [27], as evidenced by the divergent 3’ bias in the type C read density for HB_S67 (Figure 2), we suggest the small number of sequence differences in the HB_S67 assembled contigs are significantly distinct compared to those in [27] (Table S2). However, the presence of small amounts of type C in one sample is interesting but does not influence the findings of this study.

Using the NGS data, both types A and B DWV Vicuna contigs were aligned across the entire genome to look for differences that correlated with deformity. Despite this unprecedented level of detail, there were no regions of the genome where all three deformed or asymptomatic samples grouped together. Neighbor-joining trees were created to examine the phylogeny of DWV variants sequenced and assembled using three regions of the DWV genome: a 4360 bp region spanning the majority of the non-structural block including Helicase and 3C protease, and the majority of the RdRp gene (Figure 3a); and two further regions, both 145 bp in length, that represent a portion of the RdRp gene (Figure 3b and Figure S3a) and the Capsid region (Figure 3c and Figure S3b). The DWV variants are split into the three master sequences; types A, B and C. The phylogenies show that within the type A clade, deformed and asymptomatic samples from the same site never share the same dominant variant of DWV. The low amount of genetic diversity within the type B clade can be attributed to the low viral load.

Although, within each location, the sequences differed for deformed and asymptomatic samples, the variation between colonies is always greater than that within each colony i.e., deformed vs. asymptomatic. Interestingly, the asymptomatic Big Island (East) sample produced an RdRp contig which contained elements of both A and B variants (Supplementary Figure S3a, HB_S21 contig 1) indicating a possible recombination between variants. This was removed prior to creating the phylogeny (Figure 3b). Another possible A–B recombinant was also observed in the deformed Oahu sample within the Helicase gene (Figure 2). However, we could not confirm the precise recombination junction site due to the lack of specific reads covering this region.

## 4. Discussion

Although it is well known that DWV can cause wing deformity in infected individuals, the reason for those symptoms affecting only a small proportion of infected individuals remains poorly understood. Morphometric analysis carried out in this pilot study revealed wings to be either deformed or not (asymptomatic) with no intermediate phenotype. Despite a significant increase in DWV type A load detected using RT-qPCR, no significant differences in wing length were seen between bees that had been parasitised as pupae and those which had not. Furthermore, directional asymmetry is common in honey bees ([35,36,38], this study), and flies [39], and is commonly regarded as a sensitive indicator of developmental perturbation [38]. However, directional asymmetry was not affected by an increased DWV load as might be expected.

The Varroa mites’ ability to act as a vector and host of DWV [16,40], by providing an alternative transmission route; directly inoculating virus particles into the haemolymph, helps explain the higher DWV load in parasitised bees relative to non-parasitised bees [4,31]. The presence of DWV in the non-parasitised group indicates that an active non-mite transmission route must also be present, most likely via horizontal transmission (the brood food) [16] and/or by vertical transmission from queen to egg [41]. Varroa may indirectly impact this “non-mite” transmitted DWV population by increasing the amount of DWV circulating within the honey bee population. A previous study by Teixeira et al. [42], found DWV in approximately 20% of adult bees’ abdomens in Brazil, while in 2015, 100% of individuals were positive for DWV [this study]. An increase in Brazilian bees with deformed wings has not been reported, despite colonies hosting mite populations of up to 3500 [43]. Mites entering brood cells normally have very low DWV loads relative to those leaving cells 12 days later [33] potentially as a result of viral replication within the mite [15]. However, DWV is continually passed between the bee and mite during regular bouts of feeding [40] and potentially replicates in both. The host in which predominant amplification occurs remains unclear. A recent study demonstrated the absence of non-structural and high abundance of structural proteins in Varroa, suggesting that DWV proteins accumulated in the gut after feeding and not as a result of viral replication in the mite [44]. However, studies using FISH (Fluorescence in situ hybridization) probes or immunohistochemical techniques may help resolve this uncertainty. At the present time, it is difficult to say whether the high viral load in bees is a symptom of being parasitised by a mite carrying a high viral load, or if the mites have a high viral load because of a high level of viral replication in the bee on which the mite is feeding. As a result, there is no clear explanation for why only a small proportion of parasitised bees develop deformed wings. A study by Bowen-Walker et al. [7] found that when transferring mites from pupae which developed deformed wings to new host pupae, the majority but not all of the new pupae went on to develop deformity. Further experiments are required to repeat this work.

We hypothesised that a specific variant within the quasispecies causes deformity through either an increased ability of specific sequences to replicate in the bee, or potential tissue tropism of certain variants. Analysis of NGS data showed there to be no consistent differences between deformed and asymptomatic bees in terms of the dominant DWV consensus genomes. In addition, inter-colony variation was always larger than intra-colony variation i.e., between deformed and asymptomatic bees. However, as previously reported [3,45], the DWV infection of deformed bees was always dominated by the type A master variant. This suggests that a “deformed phenotype variant” of type A is unlikely since it is also present in asymptomatic bees. We suggest that the alteration of the DWV variant landscape (e.g., by Varroa), which differs from that already present in the hive may result in disease progression. Recombinants of types A and B have previously been proposed to result in a virulent infection [31], however this was not seen here and has not since been shown in other populations. We did find recombinants between types A and B, however they were not dominant in the samples. Their low load, and resulting low genetic diversity, as observed in the phylogeny, indicates a low level of replication and thus virulence. Although no clear link between DWV genomes and deformity was detected, Vicuna consensus contigs within the RdRp segment were consistently different in each pair of samples from each location (Figure 3b and Figure S3a), suggesting that many variants have the potential to cause wing deformities. The Fernando de Noronha study [9] has helped support ideas that wing deformity is not caused by the direct effects of mite feeding or haemolymph extraction, and this study failed to find any unique DWV variant linked with deformity. So currently, the only consistent factor associated with deformed wings is the high DWV load, but it remains unclear if the high load causes deformity or results from another factor that initially causes the deformity. As such, future work is needed on a larger scale with the investigation of additional considerations to ascertain the influence of other factors on the development of deformity.

## 5. Conclusions

These two pilot studies aimed to ascertain whether there was a specific DWV variant within the replicating quasispecies which was associated with the development of the deformed wing phenotype. Using a combination of NGS, RT-qPCR and HRM, we confirmed that DWV type A dominated in all samples although types B and C, as well as A/B and A/C recombinants were also found to be replicating at lower levels. Significantly, there was no clustering between deformed samples and asymptomatic samples, indicating that no unique DWV variant is associated with wing deformity. Furthermore, we found that neither DWV load nor dominant variant correlated with wing asymmetry which might have been expected given the fact that wing asymmetry is often used as an indicator of developmental perturbation. This study indicates that no specific genomic pattern of DWV can be used in predicting wing deformities in honey bees.

## Supplementary Materials

The Supplementary materials are available online at http://www.mdpi.com/2075-4450/8/1/28/s1. Figure S1: High Resolution melt plot of DWV RdRp RT-PCR products amplified from Brazilian deformed bees (red) and asymptomatic bees from cells infested with (yellow) and without Varroa (green). All melt peaks are in the predicted DWV type A variant region (78.5°C–82°C); Figure S2: Multiple sequence alignment (Geneious 8.1.7, Biomatters) of sequenced RdRp RT-PCR products amplified from Brazilian bee samples and used for HRM analysis (Figure S1) mapped to DWV type A. Deformed bee samples are highlighted red, asymptomatic bees from cells infested with Varroa are yellow and without Varroa are green, as in Figure S1. Nucleotides that differ to the reference sequence are highlighted. Sequences are also included for the Type B and Type C genomes for comparison; Figure S3: Multiple sequence alignment of DWV Type A (NC_004830.2), B (AY251269.2) and C (ERS657949) reference sequences with contigs assembled by Vicuna in the (**a**) RdRp region and (**b**) Capsid encoding region of the DWV genome. Nucleotides that differ to the DWV type A reference sequence are highlighted. DWV type A, B and C sequences are highlighted in red, blue and yellow respectively; Table S1: The primers used for RT-qPCR in this study; Table S2: Degree of similarity calculated using a global alignment between the DWV type C genome previously described and the type C contigs assembled from sample HB_S67 in this study.
