# Supplementary material for: A Comparison of Deformed Wing Virus in Deformed and Asymptomatic Honey Bees
Source: Insects. 2017 Mar 7;8(1):28. doi: 10.3390/insects8010028 (PMC5371956; doi:10.3390/insects8010028)
Supplement: Supplementary file 1 [file insects-08-00028-s001.pdf]

Supplementary Materials: A Comparison of Deformed Wing Virus in Deformed and Asymptomatic Honey Bees

Laura E. Brettell, Gideon J. Mordecai, Declan C. Schroeder, Ian M. Jones, Jessica R. da Silva, Marina Vicente-Rubiano and Stephen J. Martin

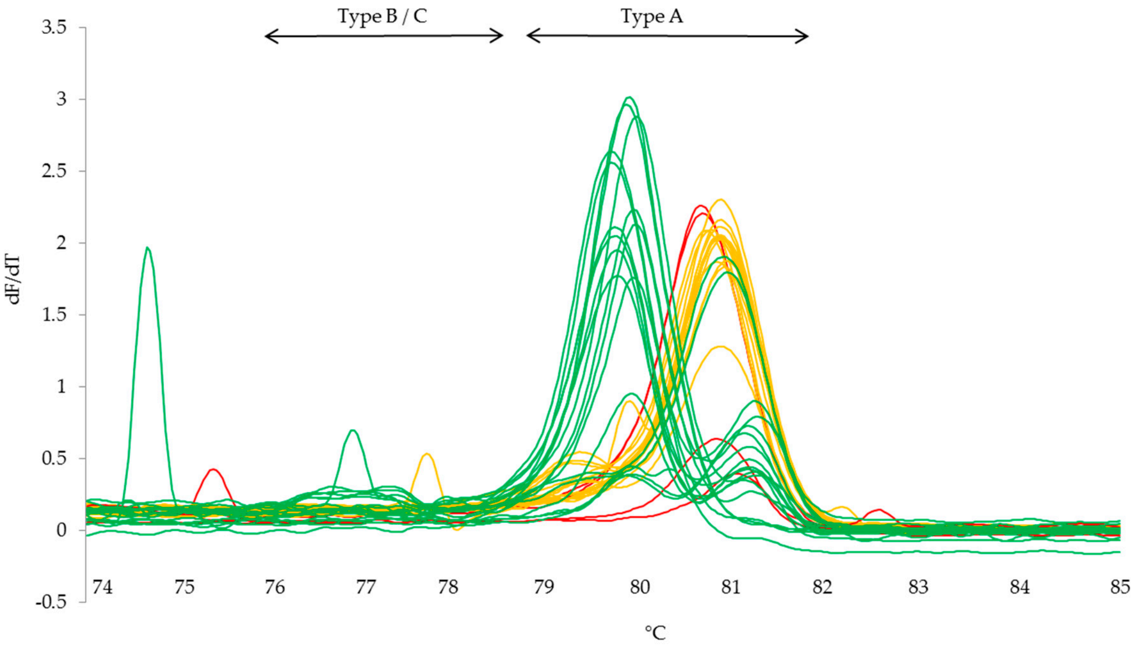

Figure S1. High Resolution melt plot of DWV RdRp [1] RT-PCR products amplified from Brazilian deformed bees (red) and asymptomatic bees from cells infested with (yellow) and without Varroa (green). All melt peaks are in the predicted DWV type A variant region (78.5°C–82°C, [2]).

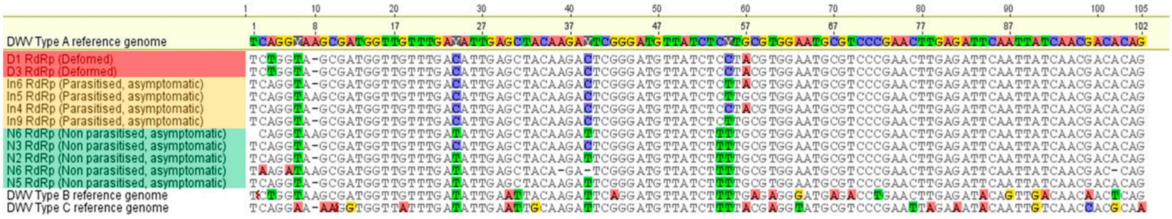

Figure S2. Multiple sequence alignment (Geneious 8.1.7, Biomatters) of sequenced RdRp RT-PCR products amplified from Brazilian bee samples and used for HRM analysis (Figure S1) mapped to DWV type A. Deformed bee samples are highlighted red, asymptomatic bees from cells infested with Varroa are yellow and without Varroa are green, as in Figure S1. Nucleotides that differ to the reference sequence are highlighted. Sequences are also included for the Type B and Type C genomes for comparison.

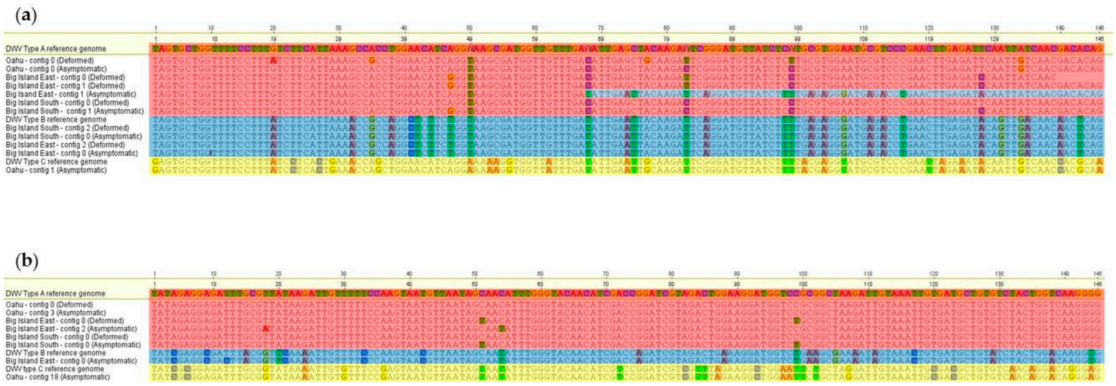

Figure S3. Multiple sequence alignment of DWV Type A (NC_004830.2), B (AY251269.2) and C (ERS657949) reference sequences with contigs assembled by Vicuna in the (**a**) RdRp region [1] and (**b**) Capsid encoding region of the DWV genome. Nucleotides that differ to the DWV type A reference sequence are highlighted. DWV type A, B and C sequences are highlighted in red, blue and yellow respectively.

Table S1. The primers used for RT-qPCR in this study, taken from Highfield et al. [1].

Target	Primer name	Sequence (5'-3')
DWV (RdRp region)	DWVQ_F1<br>DWVQ_R1	TAGTGCTGGTTTTCCTTTGTC<br>CTGTGTCGTTGATAATTGAATCTC
Actin	Actin_F1<br>Actin_R1	CCTGCAATCGCAGATAGAATGC<br>AAGAATTGACCCACCAATCCATAC

Table S2. Degree of similarity calculated using a global alignment between the DWV type C genome previously described [3] and the type C contigs assembled from sample HB_S67 in this study.

Contig #	Similarity
dg-29	99.52%
dg-10	100%
dg-8	100%
dg-14	99.33%
dg-7	100%
dg-5	100%
dg-18	100%
dg-0	99.38%
dg-3	99.72%
dg-1	99.91%

generated by VICUNA.

References

1. Highfield, A.C.; El Nagar, A.; Mackinder, L.C.M.; Noel, L.M.-L.J.; Hall, M.J.; Martin, S.J.; Schroeder, D.C. Deformed Wing Virus Implicated in Overwintering Honeybee Colony Losses. *Appl. Environ. Microbiol.* **2009**, *75*, 7212–7220.
2. Mordecai, G.J.; Brettell, L.E.; Martin, S.J.; Dixon, D.; Jones, I.M.; Schroeder, D.C. Superinfection exclusion and the long-term survival of honey bees in Varroa-infested colonies. *ISME J.* **2016**, *10*, 1182–1191.

3. Mordecai, G.J.; Wilfert, L.; Martin, S.J.; Jones, I.M.; Schroeder, D.C. Diversity in a honey bee pathogen: First report of a third master variant of the Deformed Wing Virus quasispecies. *ISME J*. **2016**, *10*, 1264–1273.

© 2017 by the authors; licensee MDPI, Basel, Switzerland. This article is an open access article distributed under the terms and conditions of the Creative Commons by Attribution (CC-BY) license (http://creativecommons.org/licenses/by/4.0/).
